# Photo-Induced Vertical Alignment of Liquid Crystals via In Situ Polymerization Initiated by Polyimide Containing Benzophenone

**DOI:** 10.3390/polym9060233

**Published:** 2017-06-18

**Authors:** Fei Wang, Leishan Shao, Qiyao Bai, Xinyuan Che, Bin Liu, Yinghan Wang

**Affiliations:** 1 State Key Laboratory of Polymer Materials Engineering of China; College of Polymer Science and Engineering, Sichuan University, Chengdu 610065, China; 2015323090041@stu.scu.edu.cn (F.W.); Daisy9596@163.com (Q.B.); cxy2110@163.com (X.C.); 13208180271@163.com (B.L.); 2 Research Institute of Maoming Petrochemical Company, SINOPEC, Maoming 525021, China; shaoleishan@163.com

**Keywords:** vertical alignment, photopolymerization, alignment stability, surface free energy

## Abstract

Vertical alignment of liquid crystal (LC) was achieved in an easy and effective way: in situ photopolymerization of dodecyl acrylate (DA) monomers initiated by polyimide based on 3,3′,4,4′-benzophenonetetracarboxylic dianhydride and 3,3′-dimethyl-4,4′-diaminodiphenyl methane (BTDA-DMMDA PI). The alignment behavior and alignment stabilities were characterized by a polarizing optical microscope (POM), which showed a stable vertical alignment after 12 h of thermal treatment. The chemical structures, morphology, and water contact angles of alignment films peeled from LC cells with and without DA monomers were analyzed by means of a Fourier transform infrared spectrometer (FTIR), a scanning electron microscope (SEM), and a contact angle tester, separately. The results confirmed that the DA monomers underwent self-polymerization and grafting polymerization initiated by the BTDA-DMMDA PI under ultraviolet irradiation, which aggregated on the surfaces of PI films. The water contact angles of the alignment films were about 15° higher, indicating a relative lower surface energy. In conclusion, the vertical alignment of LC was introduced by the low surface free energy of PI films grafted with DA polymer and intermolecular interactions between LC and DA polymers.

## 1. Introduction

A liquid crystal (LC) alignment layer is a crucial component of liquid crystal displays (LCDs), which has a great influence on the LCDs’ optical and electrical performance in terms of view angles, response time, and voltage holding ratio, among others. Pretilt angle and anchoring energy governed by the chemical and topological structures of alignment layers are two critical factors in adjusting the characters of LCDs. The vertical alignment mode (VA mode), which needs a pretilt angle above 88° has many advantages, including a high on-axis contrast ratio, a wide viewing angle, satisfactory cost, and simultaneous applicability of reflective and transmissive mode over other alignment modes such as twist nematic mode and in-plane switching mode [1,2]. Therefore, VA mode has received much research attention and has been adopted into many types of LCDs, ranging from minor-sized cell phones to large-sized televisions and other devices [3].

In order to achieve perfect vertical alignment, many methods have been taken to control the pretilt angle, such as rubbing vertical alignment [4,5,6,7,8,9], polymer-sustained vertical alignment (PSVA) [10,11,12], and photo-induced vertical alignment [13,14,15,16,17]. Among these alignment methods mentioned above, the PSVA technology showing strength through its fast response, high transmittance, and simple manufacturing process [18] has been widely investigated. Generally, this technology is conducted as follows: the LC cell containing LC and UV-curable monomers is UV-irradiated under a voltage larger than the Freedericksz transition voltage, and the pretilt angle is fixed by the polymer networks formed during UV irradiation. Improved electro-optical properties and image quality, with a higher light transmittance, a lower rising time, and a lower operating voltage, were reported in PSVA LCDs. Many kinds of UV-curable monomers were used to realize PSVA, including reactive mesogen [1,19,20,21], long alky monoene, and polyene [22,23]. In the meantime, photoinitiators were added to obtain a fast reaction rate. However, the photoinitiators became impurity ions resulting in image sticking when they remained after photopolymerization [24]. The UV-curable monomers polymerized without additional initiators, such as 4,4′-diacryloyloxybiphenyl [12] and phenanthrene-carrying monomers [25], were used, aimed at solving the above problem as reported previously. Furthermore, Kang et al. [26] discovered that the pretilt angle of homogeneous alignment polyimide (PI) film was controlled using photocurable monomer (NOA65) without photoinitiator. Inspired by these results, we proposed using photosensitive PI as a photoinitiator to initiate long alkyl monoene to obtain a uniform and stable vertical alignment.

Benzophenone (BP) is an efficient photoinitiator and has been adopted for surface grafting modification through hydrogen abstracting [27,28,29]. Further, Yu et al. [30] found that the PI containing BP groups could induce homogeneous alignment of LC after polarized UV irradiation via intermolecular crosslinking initiated by BP. Therefore, the PI containing BP group could be targeted as polymer initiator. In addition, in order to obtain a higher reaction rate, the diamine 3,3′-dimethyl-4,4′-diaminodiphenyl methane (DMMDA) was used as a hydrogen donor for photoinitiating.

In this work, the PI (BTDA-DMMDA PI) synthesized through the polycondensation of 3,3′,4,4′-benzophenonetetracarboxylic dianhydride (BTDA) and DMMDA served as a polymer photoinitiator, and dodecyl acrylate (DA) was grafted onto the PI film to generate a uniform vertical alignment. Furthermore, the chemical structure and morphology of the PI films peeled from LC cells with and without DA monomers, as well as the alignment behavior and its thermal stability, were characterized and analyzed. This will provide the vertical alignment method with a simple procedure and free of additional micromolecular initiators.

## 2. Materials and Methods

### 2.1. Materials

3,3′-dimethyl-4,4′-diaminodiphenyl methane (DMMDA) and 4,4-diaminodiphenyl ether (ODA) were purchased from Shanghai EMST Corp. (Shanghai, China) and used after recrystallization from ethanol. 3,3′,4,4′-benzophenonetetracarboxylic dianhydride (BTDA) and 4,4′-Oxydiphthalic anhydride (ODPA) (>98%, Shanghai Research Institute of Synthetic Resins, Shanghai, China) were purified by a recrystallization from acetic anhydride. *N*-methyl-2-pyrrolidone (NMP, electronic grade, 99.9%) and DA were obtained from Aladdin and used as received without further purification. Negative LC (no = 1.4748, Δ*n* = 0.083, Δε =−4.2, TN-L = 90 °C) was supplied by Yantai Xianhua Chem-Tech Co., Ltd. (Yantai, China).

### 2.2. Synthesis of Poly(Amic Acid)

Polyimide (PI) was prepared via a typical two-step method with a synthesis of poly(amic acid) (PAA) and subsequent thermal imidization. Specifically, 1.00 mmol DMMDA was charged into a 50 mL three-necked flask, and 4.94 g of NMP was added to dissolve DMMDA under magnetic stirring. Exactly 1.00 mmol BTDA was added after DMMDA dissolved completely. The reaction was conducted under N_2_ atmosphere for 4 h at room temperature in order to obtain the viscous PAA solution. Subsequently, another 5.48 g of NMP was added to dilute the PAA solution to 5 wt % to obtain proper viscosity for spin-casting on ITO glass.

The polycondensation of BTDA and ODA as well as ODPA and ODA was obtained in the same manner that poly(amic acid) based on 3,3′,4,4′-benzophenonetetracarboxylic dianhydride and 4,4-diaminodiphenyl ether (BTDA-ODA PAA) and poly(amic acid) based on 4,4′-Oxydiphthalic anhydride and 4,4-diaminodiphenyl ether (ODPA-ODA PAA) were obtained.

### 2.3. Preparation of Liquid Crystal Cells

The ITO glass was washed with 3 wt % NaOH aqueous, detergent, and alcohol successively and dried at 120 °C in an oven for 3 h. The PAA solution was spin-coated onto the ITO glass at a rotation speed of 600 rpm for 9 s and 2500 rpm for 30 s. Then, the coated ITO glass was heated on a plate heater at 80 °C for 30 min, 120 °C for 30 min, 180 °C for 30 min, and 230 °C for 1 h in turn to achieve imidization of polyimide based on 3,3′,4,4′-benzophenonetetracarboxylic dianhydride and 3,3′-dimethyl-4,4′-diaminodiphenyl methane (BTDA-DMMDA PI) films. Two pieces of coated ITO glass were rubbed with a rubbing machine (TianLi Co. Ltd., Guangdong, China) and assembled in the antiparallel rubbing direction with a cell gap of 40 μm, which was set by an adhesive film spacer. DA was mechanically mixed with LC at weight ratios of 2/98, 1/99, 0.5/0.95, and 0/100 under magnetic stirring for 4 h at room temperature. The mixtures were charged into cells by a capillary action at 95 °C on a plate heater, and the cells were maintained at 95 °C for another 20 min to eliminate the flow effect. The cells were radiated with unpolarized UV light (OSRAM 300 W, ORSAM, Munich, Bayern, Germany) for 0.5 h and the distance between light and cells was 10 cm.

The cells containing polyimide based on 3,3′,4,4′-benzophenonetetracarboxylic dianhydride and 4,4-diaminodiphenyl ether (BTDA-ODA PI) and polyimide based on 4,4′-Oxydiphthalic anhydride and 4,4-diaminodiphenyl ether (ODPA-ODA PI) were prepared with the same procedures. To be emphasized, the BTDA-ODA PI and ODPA-ODA PI films were not rubbed for easy observation of alignment condition.

### 2.4. Characterization

The alignment performance of LC was characterized by polarizing optical microscope (POM) (Shanghai Millimeter Precision Instrument Co. Ltd. (Shanghai, China)) and pretilt angle tester (Changchun Institute of Optics, Fine Mechanics and Physics (Changchun, China)). The Fourier transform infrared (FTIR) spectra of alignment layers were recorded with Nicolet 560 FTIR spectrometer (Thermo Nicolet Corporation, Madison, WI, USA) to determine the chemical structures. Scanning electron microscopy (SEM) photographs were taken with a Quanta 250 scanning electron microscope (FEI, Hillsboro, OR, USA) under an acceleration voltage of 20 kV to characterize the surface morphologies of alignment layers. The alignment layers were carefully peeled off from cells with a process of being soaked in acetone and deionized water for 30 min each and washed several times to completely remove LC, unreacted monomers, and homopolymers prior to FTIR testing. The alignment layers peeled off from cells were named DA-0.5, DA-1, and DA-2, separately, while DA-0 referred to the BTDA-DMMDA PI film, shown in Table 1. The contact angles of the alignment layers were measured by a contact angle-meter (DSA100, Kruss, Hamburg, Germany), and the total surface free energy was calculated with Method-EOS. The cells were disassembled to test the SEM and water contact angles via bath and washing with acetone, i.e., the alignment layers still adhered to the glass. The contrast ratios of LC cells were determined by a ZKY-LCDEO-2 liquid crystal electro-optic effect comprehensive tester (Chengdu century Zhongke Instrument Co., Ltd., Chengdu, China). The cells were UV irradiated and thermal annealing at 120 °C for 30 min before the test. For comparison, a regular PSVA mode cell supplied by Yantai Xianhua Chem-Tech Co., Ltd., (Yantai, China) was determined using the same method.

## 3. Results and Discussion

### 3.1. Analysis of Chemical Structures of Polyimide

**Figure 1:**
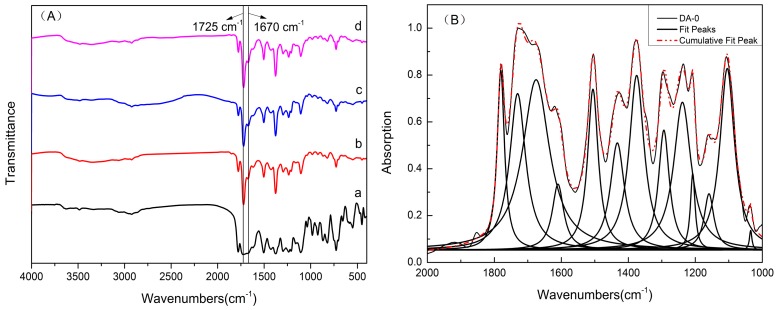
(**A**) FTIR spectra of polyimide based on 3,3′,4,4′-benzophenonetetracarboxylic dianhydride and 3,3′-dimethyl-4,4′-diaminodiphenyl methane (BTDA-DMMDA PI) films peeled from liquid crystal (LC) cells with different weight ratios of dodecyl acrylate (DA) monomers: a: DA-0, b: DA-2, c: DA-1, and d: DA-0.5; (**B**) FTIR spectrum a (black line) and its peek fitted with Lorentzian functions curves (red dash line and bold black lines).

FTIR is useful for comparing the chemical structures of alignment layers before and after UV photo radiation. As shown in Figure 1A, spectrum a showed the characteristic absorption peaks of initial BTDA-DMMDA PI (DA-0) without photo radiation at 1779, 1727, and 1376 cm^−1^, ascribed to the symmetric stretching vibration of C=O, the asymmetric stretching vibration of C=O, and the stretching vibration C–N in imide groups, separately [31]. The peak of C=O in BP providing photoinitiating sites was located at 1670 cm^−1^. The breathing vibration of aromatic rings near 1505 cm^−1^ [32] remained the same before and after UV irradiation, which was a proper internal standard for measuring the reaction degree of C=O in BP.

It is well known that the BP initiates free radical polymerization through hydrogen abstracting as mentioned above. Hydrogen abstracting will break the C=O in BP and generate –OH [30]. Therefore, as shown in Figure 1A, –OH stretches (weak) appeared at 3342 cm^−1^, and the peak intensity of C=O in BP at 1670 cm^−1^ decreased after photoirradiation, which provided support for photoinitiating C=O in BP. The peak intensity of C=O at 1721 cm^−1^ was increased after UV irradiation, due to the grafting of DA. Previous work [33] reported that the peak could be fitted with Lorentzian functions to estimate the reaction degree of C=O in BP. Similarly, the peaks of FTIR spectra were fitted with Lorentzian functions prior to comparing the amounts of grafting of DA between cells containing different weight ratios of DA monomer as shown in Figure 1B. The values of *S*_1720_/*S*_1505_ and *S*_1670_/*S*_1505_ were displayed in Table 1. The PI in four LC cells added with different weight ratios of DA showed different intensity increase ratios of the peak centered at 1721 cm^−1^ and intensity decrease ratios of C=O in BP. Generally, the peak at 1721 cm^−1^ increased and the peak at 1670 cm^−1^ decreased with the increase in DA monomer weight ratios. This may have resulted from the increased reaction probabilities of DA monomers grafted onto PI films in LC cells with more weight ratios of DA.

### 3.2. Alignment Behavior of Liquid Crystals

**Figure 2:**
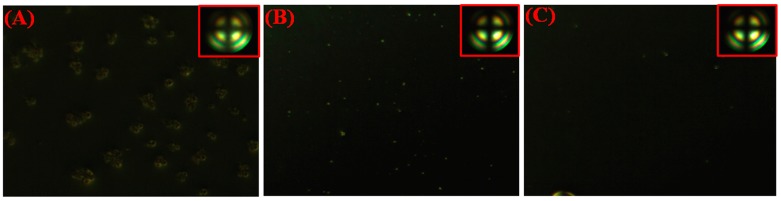
Polarizing optical microscope (POM) microphotographs of LC cells with 2 wt % DA monomers (**A**), 1 wt % DA monomers (**B**), and 0.5 wt % DA monomers (**C**) after UV irradiation.

The orthoscopic and conoscopic (inset) POM graphs of LC cells with different weight ratios of DA were shown in Figure 2. The LC cells with 2%, 1%, and 0.5% of DA showed dark state under orthogonal polarization after photoirradiation. Moreover, a dark cross brush in the conoscopic POM graphs indicated that the LC aligned vertically. The pretilt angles were 89.7° of cells with DA monomers, which also demonstrated vertical alignment. However, the cell with 2% DA showed some light spots, which may be due to the incomplete phase separation of DA homopolymers in LC [34]. Compared with the cell with 2% DA, the cells with 1% DA and 0.5% both revealed a much better dark state. This is likely owing to the less self-polymerized DA polymers in cells with a lower monomer concentration.

**Figure 3:**
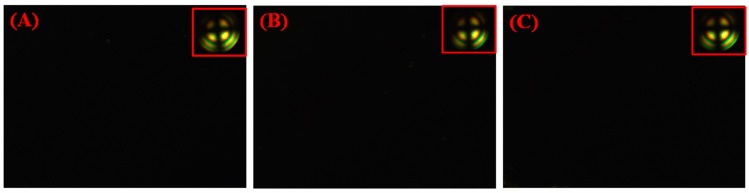
POM microphotographs of LC cells with 2 wt % DA monomers (**A**), 1 wt % DA monomers (**B**), and 0.5 wt % DA monomers (**C**) after UV irradiation and thermal annealing at 120 °C for 30 min.

Figure 3A showed that, after thermal annealing at 120 °C for 30 min, the cell with 2% DA exhibited a better dark state, proving that the light spots were caused by incomplete phase separation. In addition, the dark states of cells with 1% DA and 0.5% were also better off. Moreover, the contrast ratios of LC cells were determined. The results showed that the contrast ratios (Table 1) of the LC cells with 1% and 0.5% DA were above 400:1 and similar to the regular PSVA mode cell (408:1). The LC cell with 2% DA had a relative lower contrast ratio, which may be due to the scattering of DA polymer protrusions. This was consistent with the POM results.

**Figure 4:**
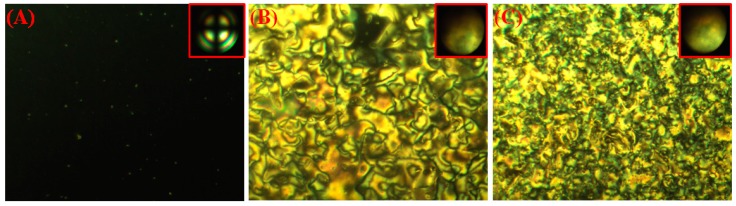
POM microphotographs of LC cells comprising BTDA-DMMDA PI film (**A**), polyimide based on 3,3′,4,4′-benzophenonetetracarboxylic dianhydride and 4,4-diaminodiphenyl ether (BTDA-ODA PI )film (**B**), and polyimide based on 4,4′-Oxydiphthalic anhydride and 4,4-diaminodiphenyl ether (ODPA-ODA PI) film (**C**) with 1 wt % DA monomers after UV irradiation.

**Figure 5:**
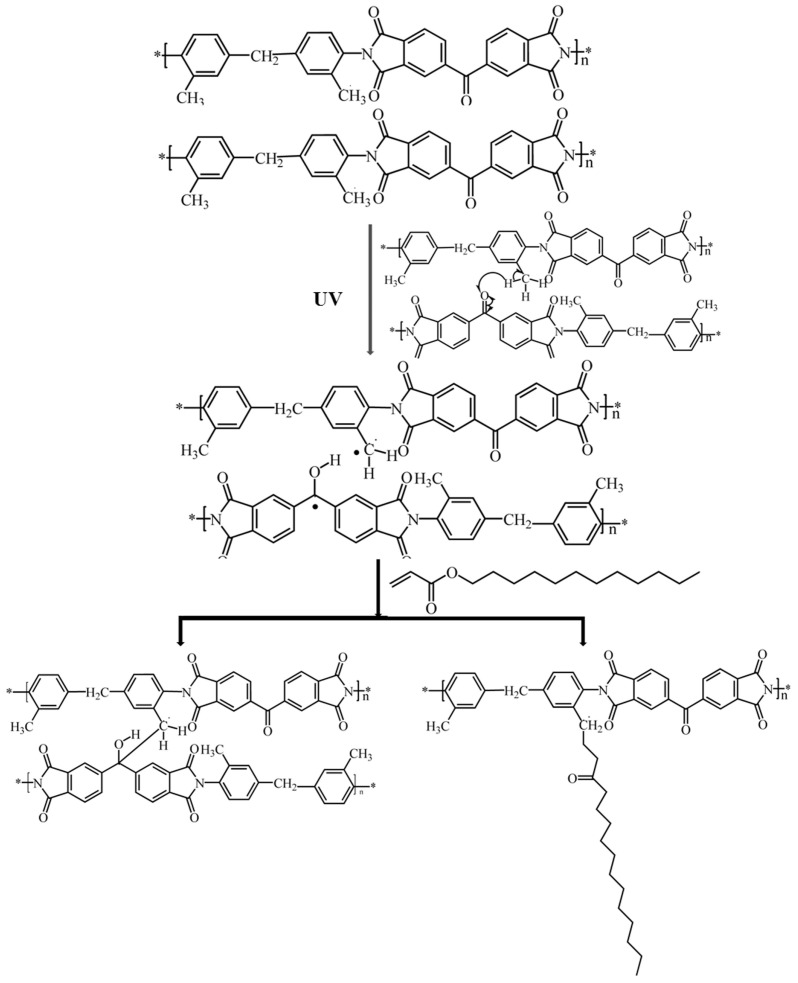
Scheme of the photoreaction of BTDA-DMMDA PI and DA monomers.

In order to reassure that the BTDA-DMMDA PI could initiate the photopolymerization of DA, two contrast experiments by varying the structure of diamine and dianhydride have been carried out. The DA monomers in cells with BTDA-ODA PI films and ODPA-ODA PI films scarcely polymerized, as the POM graphs showed no signs of vertical alignment after the same period of photoirradiation, as shown in Figure 4. This was probably due to the UV absorption by PI films and the lack of initiator. These results further prove that the monomers were initiated by BTDA-DMMDA PI, and the initiating process was consistent with previous reports [28,29], as shown in Figure 5. The reactions in and on BTDA-DMMDA PI were the intermolecular crosslinking and grafting of DA. Taking the above facts, the POM photographs, and FTIR spectroscopy results into account, the conclusion that the DA monomers were mainly photoinitiated by BTDA-DMMDA PI and grafted onto it was drawn, which was in concurrence with self-polymerization in minor portions.

### 3.3. Thermal Stability of Alignment

**Figure 6:**
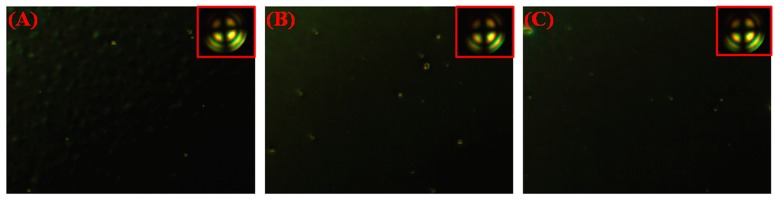
POM microphotographs of LC cells with 2 wt % DA monomers (**A**), 1 wt % DA monomers (**B**), and 0.5 wt % DA monomers (**C**) after UV irradiation and thermal annealing at 120 °C for 12 h.

Because the running of LCDs was an exothermic process, the thermal stability of alignment films should be studied. The LC cells were heated on the plate heater at 120 °C for 12 h to check the thermal stability of the vertical alignment, and the POM graphs of the heated cells were recorded and are shown in Figure 6. The POM graphs of all LC cells with different DA weight ratios exhibited a satisfactory dark state without disorders of LC after 12 h of thermal heating, which indicated satisfactory thermal stabilities. However, the cell with 2 wt % DA monomers (Figure 6A) showed small protrusions, which probably resulted from the complete phase separation and aggregation of copolymerized DA monomers. In comparison to cells with 2 wt % DA monomers, the cells with 1 wt % and 0.5 wt % DA monomers exhibited a smooth surface. This fact provided further evidence for the speculation that the incomplete phase separation was reasonably responsible for the light spots in cells containing 2% DA before thermal annealing (Figure 2A).

### 3.4. Surface Morphology of Alignment Layers

**Figure 7:**
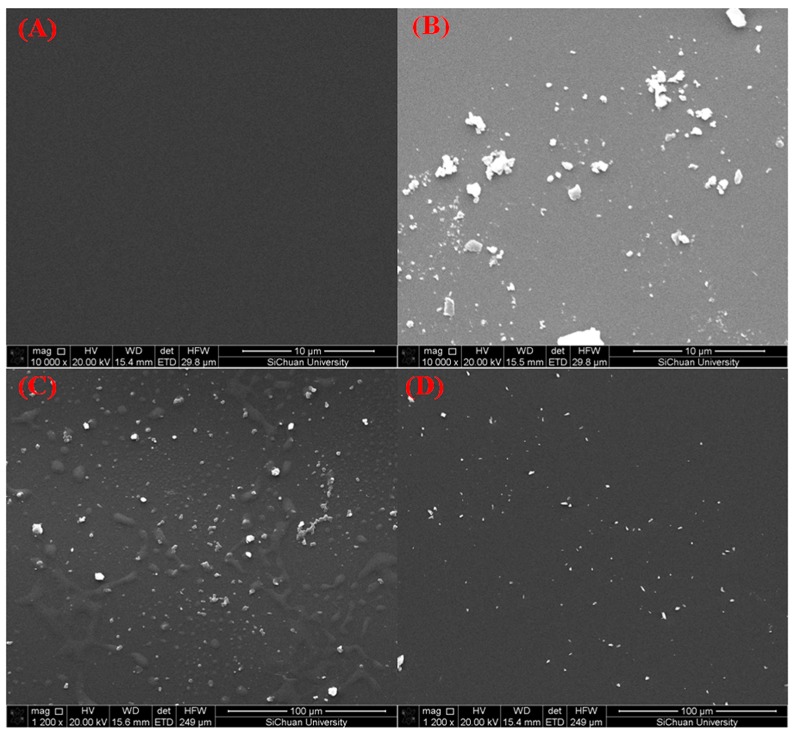
SEM photographs of PI films from LC cells with 0 wt % DA monomers (**A**), 2 wt % DA monomers (**B**), 1 wt % DA monomers (**C**), and 0.5 wt % DA monomers (**D**) after UV irradiation and thermal annealing at 120 °C for 30 min.

SEM is an effective method for investigating the morphology of alignment films. The alignment films (bottom one) from disassembled LC cells, and the LC and DA homopolymers were removed before the test. The SEM graphs of different cells were depicted in Figure 7. In order to obtain a clear overview of DA polymers grafted onto the BTDA-DMMDA PI films, 1200 magnification was used to DA-1 (Figure 7C) and DA-05 (Figure 7D). The alignment films in the cells without DA monomers showed smooth surfaces; comparatively, films in cells with DA showed surfaces that were quite rough and with many grains [22]. Moreover, the films from cells with different weight ratios of DA monomers showed different polymer particles densities. The polymer particles on alignment layers DA-2 and DA-1 were aggregated and larger. By contrast, particles on alignment DA-0.5 were dispersive and smaller. Overall, the polymer particles became larger and more intensive with the increase in the weight ratios of the DA monomers in cells. Consequently, the particles were probably DA-grafted polymers, which were inclined to aggregate on the PI films.

### 3.5. Contact Angles of Polyimide Alignment Layers

**Figure 8:**
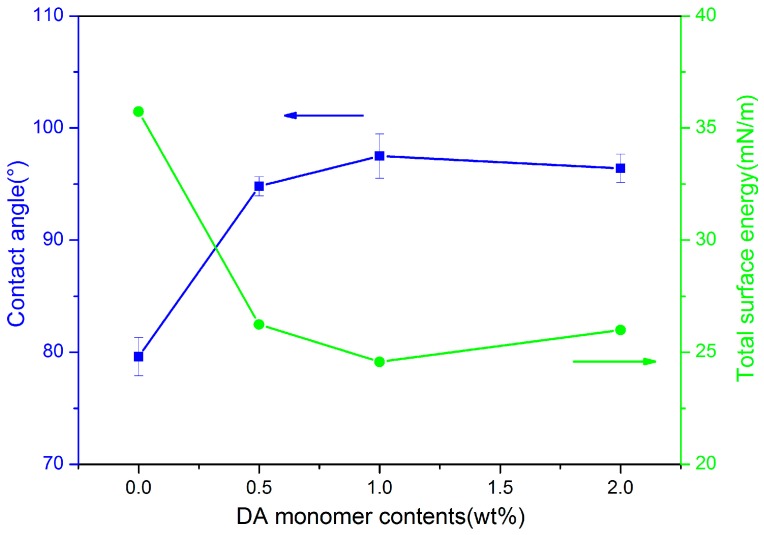
Water contact angles and total surface free energy as functions of DA monomer contents.

The surface wettability, shown in Figure 8, was determined by a contact angle tester to give a preliminary explanation for the mechanism of the vertical alignment. The contact angle of the film without photoirradiation was 79.6°, which showed homogeneous alignment after rubbing. In contrast, the BDTA-DMMDA PI films from cells with DA monomers exhibited relatively high contact angles and the specific values of DA-2, DA-1, and DA-0.5 were 96.4°, 97.5°, and 94.8°, separately. The high contact angles were induced by the DA polymers grafted onto the PI film, which made the surface quite rough and hydrophobic. Furthermore, the total surface free energies calculated with method-EOS exhibited that the intact PI film took on the highest total surface free energy of 35.73 mN/m. This result revealed that the DA monomers grafted onto the PI film caused an elevation of contact angles and the reduction of total surface free energy, contributing to the vertical alignment of LC [26,33]. In addition, the LC molecules with a rod-like shape bear alkyl groups at one end and benzene rings at the other end. Therefore, the DA polymers with long alkyl groups were able to interact with the alkyl groups of the LC molecules, which was another important factor leading to vertical alignment.

## 4. Conclusions

Vertical alignment of LC was easily achieved through in situ photopolymerization of dodecyl acrylate (DA) monomers initiated by BTDA-DMMDA PI. The dark state and dark cross brush under orthogonal polarized and conoscopic microscope separately were clearly observed, indicating vertical alignment. In addition, the vertical alignment remained stable after 12 h of thermal treatment. The morphology and chemical structures of PI films peeled from cells with and without DA monomers revealed grafting and self-polymerizing of DA monomers. In addition, the contrast experiments with different dianhydrides and diamines also provided support for grafting DA monomers onto BTDA-DMMDA PI. The vertical alignment induced by the DA-grafted PI films, with low surface free energy on the surface of the PI films, which was easily obtained and thermal stable, was a novel method adopted for vertical-alignment-mode LCDs without extra small molecular photoinitiators.

## Figures and Tables

**Table 1:** Intensities variation of carbonyl groups before and after photoirradiation and contrast ratios of LC cells.

Samples	DA-0	DA-0.5	DA-1	DA-2
PI	BTDA-DMMDA PI			
Weight ratios of DA (wt %)	0.0	0.5	1.0	2.0
S_1720_/S_1505_^a^	1.48	2.83	2.88	3.17
S_1670_/S_1505_	2.69	1.99	1.68	1.13
Contrast ratios	ND ^b^	412:1	423:1	380:1

^a^ *S* was denoted as the peak areas; ^b^ the datum was not detectable.
